# lncRNA SNHG7 sponges miR‐425 to promote proliferation, migration, and invasion of hepatic carcinoma cells via Wnt/β‐catenin/EMT signalling pathway

**DOI:** 10.1002/cbf.3429

**Published:** 2019-09-02

**Authors:** Xuebing Yao, Chi Liu, Cuiyun Liu, Wenna Xi, Shuilin Sun, Zhen Gao

**Affiliations:** ^1^ Department of Infectious Diseases Second Affiliated Hospital of Nanchang University Nanchang Jiangxi China; ^2^ Department of Medical and Life Science Chengdu University of TCM Chengdu Sichuan China

**Keywords:** epithelial‐mesenchymal transition, hepatic carcinoma, migration, miR‐425, SNHG7

## Abstract

Increasing evidence has indicated the important roles of long noncoding RNA small nucleolar RNA host gene 7 (SNHG7) in tumourigenesis as a potential oncogene. However, the function of SNHG7 in hepatic carcinoma remains unclear. In the present study, we found that SNHG7 expression was significantly upregulated in hepatic carcinoma tissues, especially in aggressive cases, and it was closely correlated with the poor prognosis. Furthermore, knockdown of SNHG7 inhibited the proliferation, migration, and invasion of hepatic carcinoma cell lines in vitro. Mechanistically, SNHG7 directly interacted with miR‐425 as a ceRNA. Moreover, knockdown of SNHG7 significantly inhibited the tumorigenic Wnt/β‐catenin/EMT pathway. SNHG7 regulated Wnt/β‐catenin/EMT pathway through sponging miR‐425 and played an oncogenic role in hepatic carcinoma progression. Together, our study elucidated the role of SNHG7 as a ceRNA in hepatic carcinoma, provided new potential diagnosis and therapeutic application in hepatic carcinoma progression.

**Significance of the study:**

SNHG7 could promote proliferation and metastasis of hepatic carcinoma cell in vitro and in vivo, suggesting that SNHG7 exerts tumorigenic role in hepatic carcinoma progression. Further mechanism research revealed that SNHG7 exhibited the tumorigenic role through Wnt/β‐catenin/EMT pathway as a miR‐425 sponge. These findings provided new cues to understand the molecular signalling network in carcinogenesis of hepatic carcinoma, and it may provide new evidence for therapeutic application in hepatic carcinoma.

## 1 INTRODUCTION

Hepatic carcinoma has been considered the fifth most common malignant cancer and third mortality rate among cancers in the world with about 1 million new cases diagnosed annually.^1^ Most patients with hepatic carcinoma die from the high rates of invasion, metastasis, and post‐operative recurrence.^2^ However, the aetiology and exact molecular mechanism on underlying hepatic carcinoma progression have not been comprehensively illuminated yet.^3^, ^4^ The carcinogenesis of hepatic carcinoma is considered to be a multifactor, multistage complex process.^5^

Recently, noncoding RNAs (ncRNAs) have caused great concerns for its complex and crucial role in cancer progression.^6^, ^7^ ncRNAs are mainly composed of small nucleolar RNAs (snoRNAs), micro‐RNAs (miRNAs), and long ncRNAs (lncRNAs). miRNA is a small ncRNA molecule consists of about 22 nucleotides. miRNA inhibits translation of mRNA by binding to the 3′ untranslated region (UTRs) of the mRNA via its seed sequence, which in turn causes degradation of mRNA. lncRNA is defined as transcripts longer than 200 nucleotides that does not translated into protein but participates in various of biological processes, including transcription regulation, posttranscriptional regulation, protein modification, and tumourigenesis especially.^8^, ^9^, ^10^ An increasing number of studies have demonstrated that lncRNA can interact with the miRNA as competing endogenous RNAs (ceRNAs) to sponges target mRNA, therefore to participate in the expression regulation of target genes that exhibit important role human diseases.^11^, ^12^

Small nucleolar RNA host gene 7 (SNHG7) is a long ncRNA with 2157 bp in length, located on chromosome 9q34.3. Recent studies have suggested that SNHG7 could promote proliferation, migration, and invasion and inhibit apoptosis in various of cancers, such as malignant pleural mesothelioma, breast cancer, colorectal cancer, and lung cancer.^13^, ^14^, ^15^, ^16^ However, the biological mechanisms and ceRNA role of SNHG7 in the tumourigenesis of hepatic carcinoma have not been elucidated. The Wnt/β‐catenin is a classical signalling pathway in regulation process of cell proliferation, differentiation, and tumourigenesis.^17^, ^18^ Epithelial‐mesenchymal transition (EMT) is regarded as the initiation phase of cancer metastasis for it involves the breakdown junctions between cells and generating individual cells with invasive capability and multiple mesenchymal attributes.^19^ Studies have shown that ncRNAs can regulate proliferation and EMT in carcinogenesis by regulating Wnt/β‐catenin signalling pathway.^20^, ^21^, ^22^ However, the role of Wnt/β‐catenin in hepatic carcinoma is unclear.

In present study, we first revealed that SNHG7 overexpression is a characteristic molecular change in hepatic carcinoma progression. SNHG7 knockdown in HepG2 and HCC‐LM3 cells significantly inhibited cell proliferation, migration, and invasion. In‐depth mechanism research revealed that SNHG7 acts as a ceRNA for hsa‐miR‐425‐5p (miR‐425), which sponged miR‐425 and regulated the Wnt/β‐catenin signalling pathway in hepatic carcinoma. Collectively, our data elucidated the crucial role of SNHG7 in hepatic carcinoma progression. Therefore, the SNHG7/miR‐425 axis might be a potential therapeutic target for hepatic carcinoma treatment.

## 2 MATERIALS AND METHODS

### 2.1 Clinical specimens and cell lines

The paired hepatic carcinoma tissues and corresponding relative normal tissues (n = 40) were collected after surgical resection in the Second Affiliated Hospital of Nanchang University according to its ethical and legal standards. All samples were saved in liquid nitrogen immediately after extraction until RNA was extracted. All patients signed a written informed consent. A 36‐month follow‐up survival survey was conducted, and the overall survival (OS) was defined as the interval between resection and death or the last follow‐up. The clinicopathological information of the 40 patients who received curative surgery at the Second Affiliated Hospital of Nanchang University between March 2011 and August 2014, including sex, age, tumour size, tumour depth, differentiation, T stage, lymph node invasion, and distant metastasis, were recorded. HepG2 and HCC‐LM3 cell lines used in this study were purchased from the Cell Bank of Type Culture Collection (CBTCC, Chinese Academy of Sciences, Shanghai, China) and cultured in RPMI‐1640 medium (HyClone, USA) supplemented with 10% fetal bovine serum (FBS) (Gibco, NY), 1% penicillin, and streptomycin in 5% CO2 at 37°C.

### 2.2 siRNA transfection

siRNA targeting SNHG7 (si‐SNHG7: 5′‐UUAGCAGAGUAAUUUGCACUU‐3′) and negative controls, as well as hsa‐miR‐425‐5p mimic and related negative control, were synthesized by GenePharma (Shanghai, China). Lentiviral vectors mediated miR‐425‐5p was constructed according to the protocol. Lipofectamine 2000 reagent (Invitrogen, USA) was used for cell transfection.

### 2.3 Dual‐luciferase reporter assay

The pcDNA3.1 SNHG7 wild/mutant type with/without designed miR‐425 binding sites were synthesized by GenePharma and cotransfected with miR‐425‐mimic or mimic negative control into HepG2 and HCC‐LM3 cells; 24 hours after transfection, luciferase activities were measured with a dual‐luciferase reporter gene assay system (Promega, USA).

### 2.4 Cell proliferation assay

MTT assay was performed to assess cell viability of HepG2 and HCC‐LM3 cells which were transfected with si‐SNHG7 or si‐NC. Briefly, cells were incubated in 96‐well plates for appropriate time, 20 μL of 5 mg/mL MTT was added to each well, and then the plates were incubated at 37°C for additional 2 hours. The medium was subsequently removed; 150 μL of DMSO was added to dissolve the purple‐coloured precipitates of formazan. Absorbance value was recorded at 490 nm using a Spectra MAX M5 microplate spectrophotometer (Molecular Devices, USA).

Colony formation assay was performed, briefly; HepG2 and HCC‐LM3 cells cotransfected with si‐SNHG7 or si‐NC were seeded in six‐well plates (200‐500 cells per well) and incubated for 10 days. Then, cells were fixed with methanol for 15 minutes and stained with 0.5% crystal violet solution for 20 minutes; the colonies (>50 cells) were counted manual with microscope (Zeiss, Germany).

### 2.5 Cell apoptosis assay

Apoptosis assay was performed with apoptosis detection kit (BD, USA) as protocol. Cells (2 × 10^5^ cells per well) were incubated in a six‐well plate and received different treatments for 48 hours; then cells were harvested and washed twice with ice‐cold PBS. Apoptosis detection kit was used to measure the levels of apoptosis according to manufacturer's instructions by FCM (BD, USA). Data were analysed by FlowJo.

### 2.6 Cell migration and invasion assay

Migration and invasion assay were conducted with Transwell chambers (Corning, USA) according to general procedure. Briefly, cells (4 × 10^4^) were culture in serum‐free medium in the upper chamber with (migration) or without (invasion) matrigel precoated; subsequently, medium containing 10% FBS was added to lower chamber as induction factor. Then, cells were incubated for 24 hours; the migrated cells on the surface of lower membrane were fixed with 70% methanol and stained by 0.1% crystal violet. The capacity of invasion was evaluated with amounts of invasive cells under a microscope (Zeiss, Germany) in three different fields of each filter.

### 2.7 Wound‐healing assay

The invasive capacity of cells transfected with si‐SNHG7 was assessed with wound‐healing assay. Generally, cells were cultured into 12‐well plates and incubated until 70% to 80% confluence. Then, artificial wound scratching was made with a sterile pipette tip. After incubated in serum‐free medium for appropriate time, wounds were captured with microscope (Zeiss, Germany).

### 2.8 Quantitative real‐time PCR

Total RNA of clinical tissues and hepatic carcinoma cells were extracted with TRIzol reagent (Invitrogen, USA) as protocol. Reverse Transcription Kit (Takara, Dalian, China) was used to reverse transcribe RNA to cDNA referring to the manufacturer's instructions. iCycler IQ Multi‐color Detection System (BioRad, USA) Quantitative was used to perform quantitative real‐time PCR (qRT‐PCR) assay as protocol. Primers used in this study were listed below:

SNHG7:
Forward: 5′‐GTGACTTCGCCTG TGATGGA‐3′.
Reverse: 5′‐GGCCTCTATCTGTACCTTTATTCC‐3′.
miR‐425:
Forward: 5′‐AAUGACACGAUCACUCCCGUUGA‐3′.
Reverse: 5′‐AACGGGAGUGAUCGUGUCAUUUU‐3′.

The GAPDH level was set as the normalization, and relative expression of SNHG7 and other mRNAs was calculated with the 2^−△△Ct^ method as standardized operation.

### 2.9 Western blot analysis

Forty‐eight hours after transfection, hepatic carcinoma cells were harvested and total protein was extracted with RIPA Lysate containing protease inhibitor (Beyotime, China). After the concentration of protein was determined and uniformized, protein was separated by 10% sodium dodecyl sulfate polyacrylamide gel. Subsequently, protein was transferred onto polyvinylidene difluoride (PVDF) membranes (Millipore, USA); PVDF membrane was blocked in 5% skim milk in TBST at 37°C for 1 hour. Subsequently, PVDF membranes were washed with TBST and incubated with primary antibodies (Abcam, UK) for β‐catenin (ab32572), MMP‐9 (ab38898), E‐cadherin (ab15148), N‐cadherin (ab76057), and β‐actin (ab124964) at 4°C overnight. After incubation with the relevant secondary antibodies for additional 1 hour at 37°C, enhanced chemiluminescence kit (Amersham, NJ) was used to detect the reactive bands as protocol.

### 2.10 In vivo tumour formation assay

Male BALB/c nude mice (4 wk old) from Huafukang Bioscience Co, Inc (Beijing, China) were used for xenograft model. The animal experiment was performed referring to the Use Committee for Animal Care and approved by the institutional guidelines of the Second Affiliated Hospital of Nanchang University. HepG2‐Lv‐SNHG7 or HepG2‐Lv‐NC cells (5 × 10^6^ cells per mouse) were injected into the right flank of mice; 7 days after injection, tumour size was measured simultaneously every 7 days with a simplified equation (length × width^2^ × 0.5). Mice were sacrificed after measurement for seven times, and the weight of tumour was measured. Finally, tumours were collected for further haematoxylin and eosin (H&E) and immunohistochemical evaluation. Tissues were collected, fixed in 4% paraformaldehyde, embedded in paraffin, and sectioned (5 μm thickness). After dewaxing and rehydration, the sections were stained with H&E at room temperature for 10 seconds and imaged under a light microscope (Olympus, Tokyo, Japan).

### 2.11 Immunohistochemistry assay

The tumour samples were resected from mice, fixed in 10% formaldehyde, embedded in paraffin, and sectioned (5 μm thickness). Immunohistochemical analyses was performed with anti‐Ki‐67 (ab15580) and anti‐MMP‐9 (ab38898) antibody obtained from Abcam. After stained with related secondary antibody for 1 hour at 37°C, slide was counterstained with haematoxylin for 20 to 30 seconds, then dehydrated and fixed; slide was sealed with the neutral gum. Images were taken with microscope (Zeiss, Germany).

### 2.12 Statistical analysis

All experiments were performed at least three times, and data were expressed as mean ± standard deviation as indicated. Sample *t* test or one‐way analysis of variance (ANOVA) were performed for statistical analysis, *P* < .05 was considered to be statistically significant. The statistical analysis was graphed with GraphPad.

## 3 RESULTS

### 3.1 Overexpressed SNHG7 in hepatic carcinoma tissues was associated with poor prognosis

**Figure 1:**
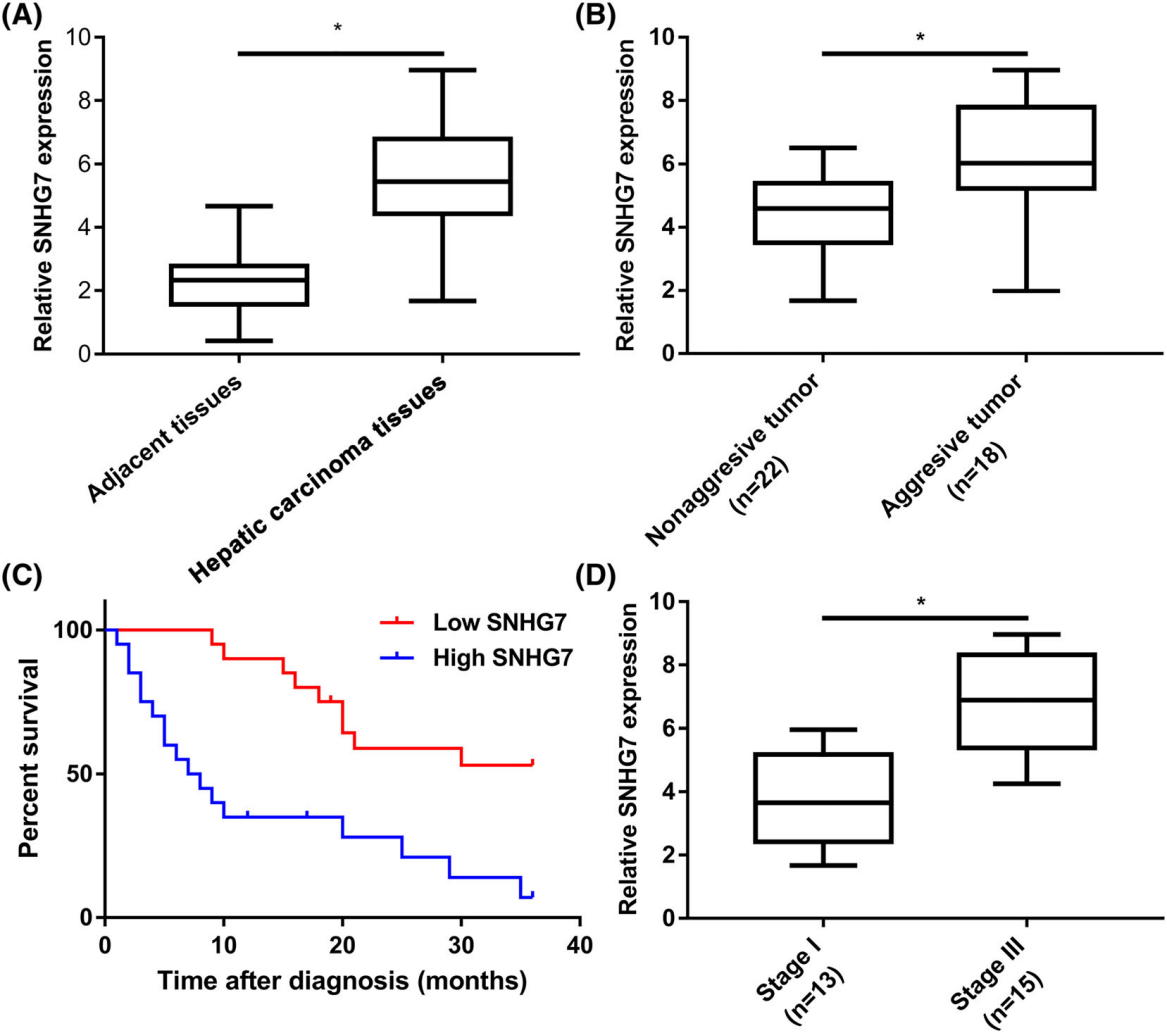
Expression level of SNHG7 in hepatic carcinoma tissues. A, Expressional profile of SNHG7 in hepatic carcinoma samples (n = 40) and related normal samples (n = 40). B, Expression of SNHG7 in hepatic carcinoma tissues of patients with metastasis (n = 22) and without metastasis (n = 18). C, Thirty‐six–month overall survival survey based on SNHG7 expression levels in 40 patients with hepatic carcinoma (*P* < 0.05; log‐rank test). The median level of SNHG7 was set as the cut‐off point. D, SNHG7 expression level in hepatic carcinoma tissues associated with TNM stage (*P* < 0.05)

To determine whether there was a difference of SNHG7 expressional profile in hepatic carcinoma and adjacent histologically normal hepatic tissues, 40 pairs of specimens were analysed with qRT‐PCR. The result suggested that SNHG7 was significantly overexpressed in hepatic carcinoma tissue compared with adjacent normal tissue (Figure 1A). Moreover, the SNHG7 expression level may be associated with the metastasis of hepatic carcinoma (Figure 1B). Further, Kaplan‐Meier survival analysis also showed that up‐regulated SNHG7 was positive correlation with poor OS of patients with hepatic carcinoma (Figure 1C). Furthermore, statistical analysis revealed that there is a significant association between overexpressed SNHG7 and bad clinical stage (Figure 1D). Overall, these findings elucidated that the overexpressed SNHG7 may be associated with progression, metastasis, and poor prognosis of patients with hepatic carcinoma.

### 3.2 Knockdown of SNHG7 suppressed cell proliferation, invasion, and migration and promoted apoptosis of hepatic carcinoma cell HepG2 and HCC‐LM3 in vitro

**Figure 2:**
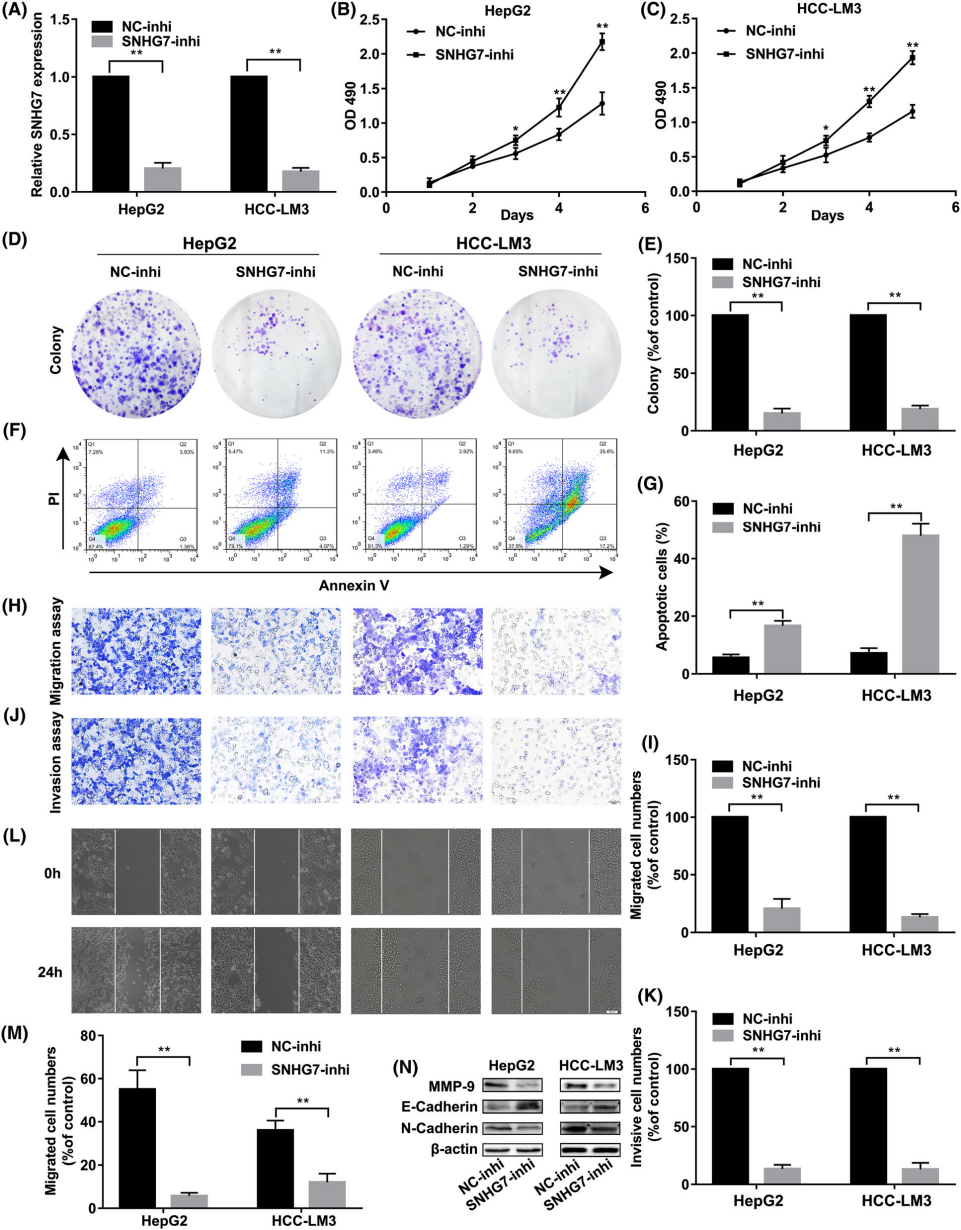
Functional analysis of SNHG7. A, SNHG7 expression level was determined by qRT‐PCR when HepG2 and HCC‐LM3 cells were transfected with SNHG7‐siRNA or NC‐siRNA. (B) Growth curves of HepG2 and (C) HCC‐LM3 cells after transfection with SNHG7‐siRNA (SNHG7‐inhi) or NC‐siRNA (NC‐inhi) were determined via MTT assays. (D,E) Colony formation assays for HepG2 and HCC‐LM3 cells transfected with SNHG7‐siRNA or NC‐siRNA. (F,G) Apoptosis level of HepG2 and HCC‐LM3 cells transfected with SNHG7‐siRNA were determined by Flow cytometry assays. (H‐K) Transwell assay was measured to determine function of SNHG7. Scale bars = 50 μm. (L,M) Migration ability of SNHG7‐siRNA and NC‐siRNA‐treated cells were assessed by Wound‐healing assay. Scale bars = 50 μm. (N) The levels of MMP‐9, E‐cadherin, and N‐cadherin following SNHG7 knockdown in hepatic carcinoma cells were assessed via western blot. *P < .05

RNA interference was performed to explore the biologic function of SNHG7 in hepatic carcinoma. siRNA targeting the coding region of SNHG7 (SNHG7‐inhi) was transfected into HepG2 and HCC‐LM3 cells, respectively. The knockdown efficiency of SNHG7‐siRNA was confirmed by RT‐PCR assay, and result showed that the expression level of SNHG7 in SNHG7‐siRNA‐transfected HepG2 and HCC‐LM3 cells was drastically decreased by 70% (Figure 2A). MTT and colony formation assay suggested that knockdown of SNHG7 significantly suppressed the proliferation of HepG2 and HCC‐LM3 cells (Figure 2B‐E). In addition, transfection of SNHG7‐siRNA induced significantly increased percentage of apoptotic cells in HepG2 and HCC‐LM3 cells (Figure 2F,G), suggesting that knockdown of SNHG7 could promote apoptosis of hepatic carcinoma. Subsequently, cell invasion and migration assays illustrated that overexpressed SNHG7 was positive correlated with migration ability of HepG2 and HCC‐LM3 cells (Figure 2H‐K). Meanwhile, wound‐healing assay suggested that knockdown of SNHG7 retarded the closing of scratch wound (Figure 2I,M). Furthermore, silence of SNHG7 led to decreased level of metastasis related protein MMP‐9 (matrix metallopeptidase 9), N‐cadherin, and increased level of cell adhesion protein E‐cadherin (Figure 2N). Above result suggested that knockdown of SNHG7 could impede hepatic carcinoma cell proliferation and metastasis.

### 3.3 SNHG7 sponging miR‐425 as a ceRNA

**Figure 3:**
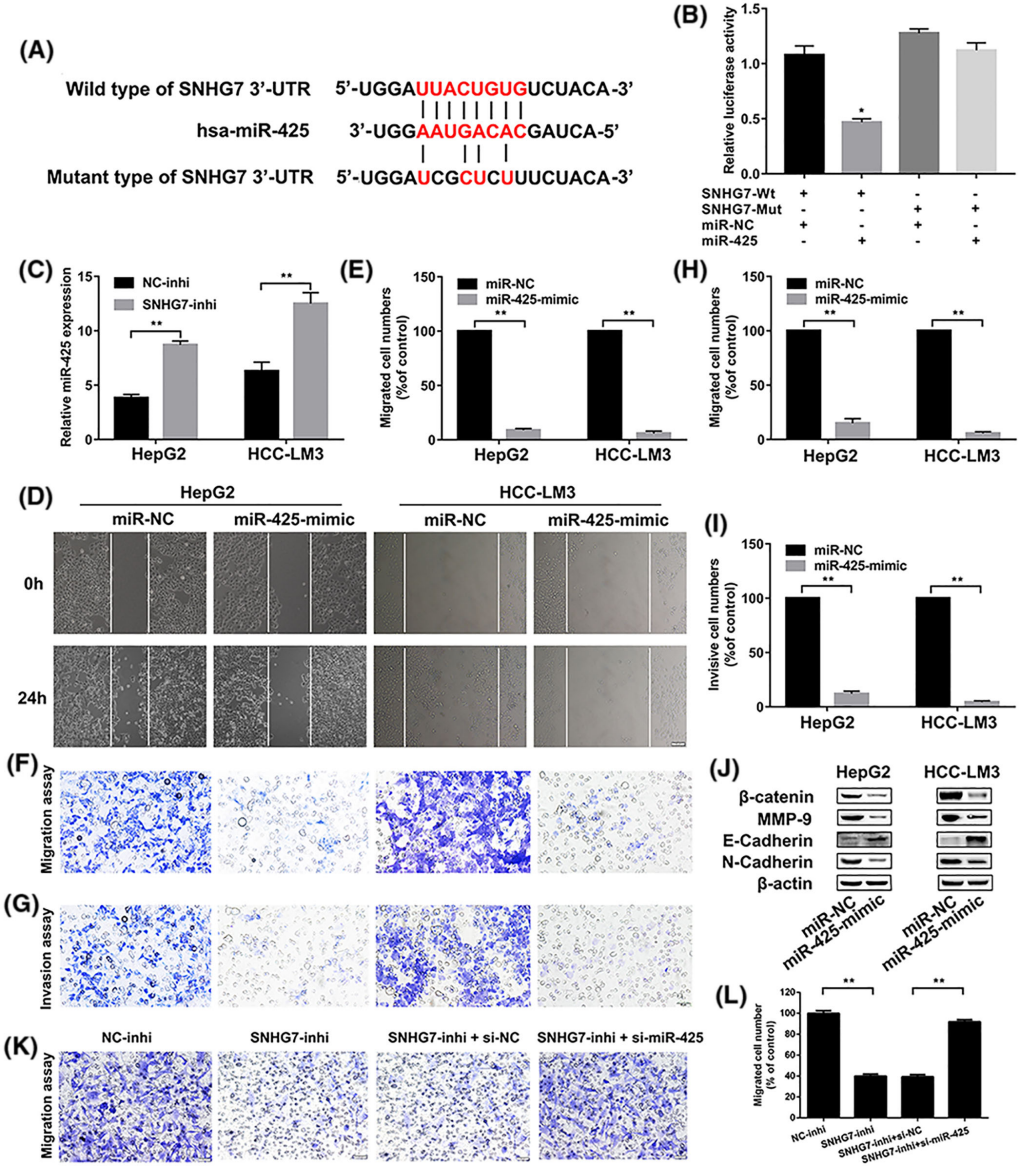
SNHG7 sponges miR‐425 to promote metastasis of hepatic carcinoma via Wnt/β‐catenin EMT signalling pathway. A, The putative binding sites of SNHG7 to miR‐425 sequence were shown. B, Luciferase activity of HepG2 cells cotransfected with luciferase reporters containing SNHG7‐Wt/SNHG7‐Mut and miR‐425 mimic/miR‐425 control were analysed with dual‐luciferase reporter assays system. C, Expression of miR‐425 was determined by qRT‐PCR when HepG2 and HCC‐LM3 cells were treated with SNHG7‐siRNA. (D,E) Migration ability of miR‐425‐mimic treated cells was assessed by wound‐healing assay. Scale bars = 50 μm. (F,G) Transwell migration assay was measured after cells were treated with miR‐425‐mimic. Scale bars = 50 μm. (H,I) Transwell invasion assay was measured after cells were treated with miR‐425‐mimic. Scale bars = 50 μm. (J) The levels of β‐catenin, MMP‐9, E‐cadherin, and N‐cadherin following miR‐425‐mimic treated in hepatic carcinoma cells were assessed through western blot. (K,L) Transwell migration assay was measured after different treatments in HepG2 cells. *P < .05

To determine whether SNHG7 could interact with the miRNA as ceRNAs or natural miRNA sponge as previous report, targetscan was conducted with bioinformatic database (TargetScanHuman Release 7.2, Starbase v3.0 and miRcode). Bioinformatics analysis indicated that there are putative binding sites between SNHG7 and miR‐425 (Figure 3A). Then dual‐luciferase reporter assay was conducted to validate the interaction between miR‐425‐5p and SNHG7 in HepG2 cells. Result revealed that luciferase activity of SNHG7‐Wt was dramatically reduced by miR‐425 mimic. In contrast, the luciferase activity of SNHG7‐Mut experienced no statistical changes (Figure 3B). These results indicated that SNHG7 could sponge miR‐425 as a ceRNA.

### 3.4 SNHG7 sponges miRNA‐425 to promote proliferation and metastasis of hepatic carcinoma cell HepG2 and HCC‐LM3 via Wnt/β‐catenin/EMT signalling pathway

miR‐425 rescue experiments were performed to explore how SNHG7 exerted its biological role through sponging miR‐425. A significant increased miR‐425 expression was observed while SNHG7 was knockdown both in HepG2 and HCC‐LM3 cells (Figure 3C). The result further confirmed SNHG7 sponging miR‐425 as a ceRNA. Then, miR‐425‐mimic was transfected into HepG2 and HCC‐LM3 cells to elucidate the function of miR‐425 in hepatic carcinoma proliferation and metastasis. Wound healing and transwell assays suggested that migratory and invasive ability were significant reduced in cells transfected with miR‐425‐mimic (Figure 3D‐I). Meanwhile, the EMT related proteins (β‐catenin, MMP‐9, E‐cadherin, and N‐cadherin) were examined. While transfected with miR‐425‐mimic, the protein levels of β‐catenin, MMP‐9, and N‐cadherin were decreased markedly. In contrast, E‐cadherin expression was increased notably both in miR‐425‐mimic transfected HepG2 and HCC‐LM3 cells (Figure 3J). Moreover, downregulation of miR‐425 could significantly block the antimigration effect, which is induced by SNHG7‐inhi treatment in HepG2 cells (Figure 3K‐L). Above results suggested that SNHG7 promotes metastasis of hepatic carcinoma cell via Wnt/β‐catenin/EMT signalling pathway as a miR‐425 sponge.

### 3.5 Knockdown of SNHG7 suppressed the growth of xenograft tumour in nude mice

**Figure 4:**
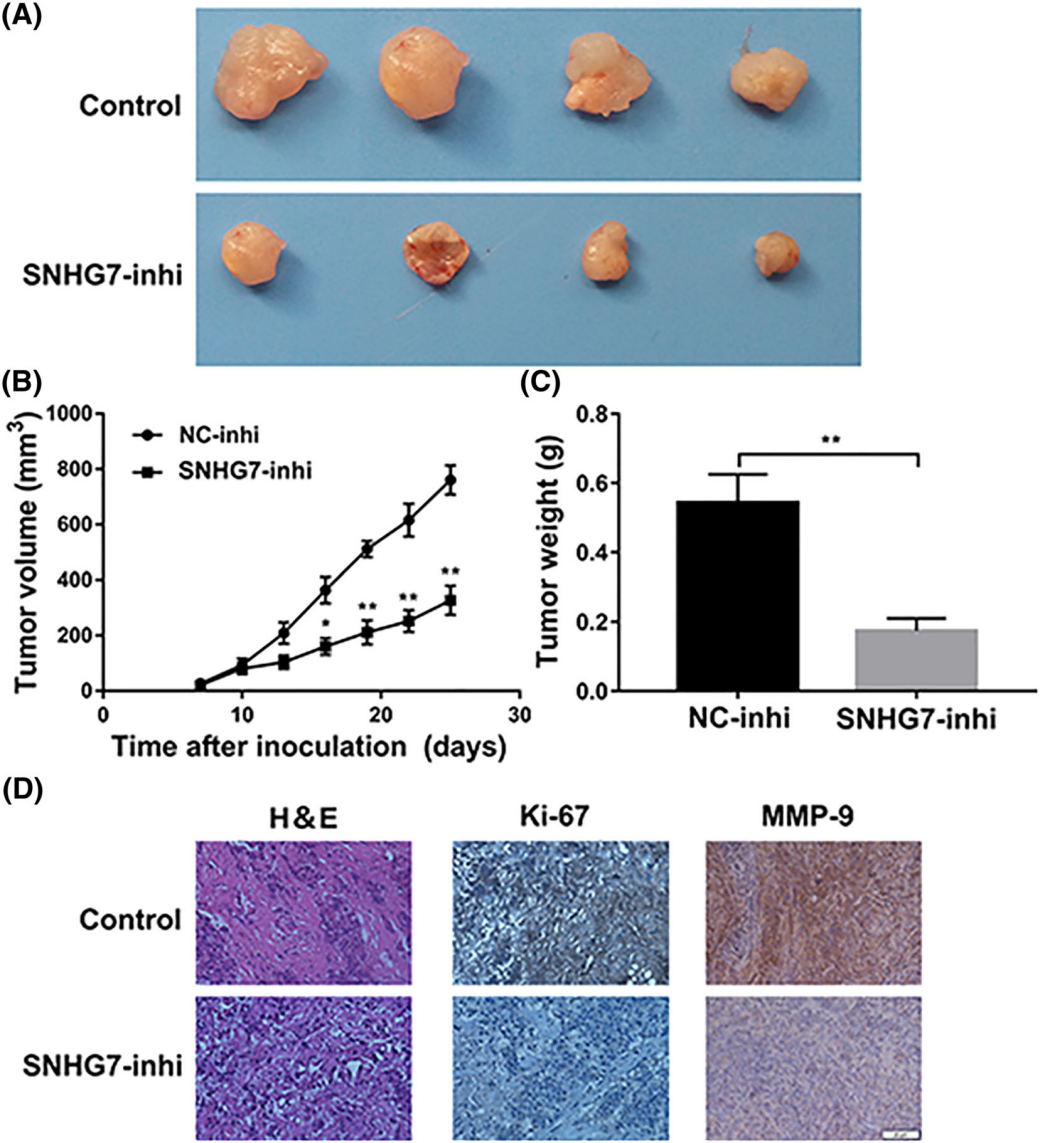
SNHG7 knockdown suppressed tumour growth in vivo. A, Tumours collected from mice. B, Tumour volume curve of mice injected with HepG2‐Lv‐SNHG7 or HepG2‐Lv‐NC cells was analysed. C, Tumour weight of mice was measured. D, Immunohistochemical staining of MMP‐9 and Ki‐67 were used to assess proliferation and metastasis

Subcutaneous xenograft tumour model in nude mice was built to study the relationship between SNHG7 and tumourigenesis in vivo. HepG2‐Lv‐SNHG7 or HepG2‐Lv‐NC cells were injected subcutaneously into the nude mice right flank. Xenograft tumour model with HepG2‐Lv‐SNHG7 cells showed obviously reduced in tumour weight and volume compared with mice HepG2‐Lv‐NC xenograft tumour model (Figure 4A‐C). Morphological changes were observed between SNHG7 silenced tumour tissues and control by H&E stain (Figure 4D). Moreover, results of immunohistochemical analysis demonstrated that SNHG7 promoted cell proliferation in tumour tissues (Ki‐67, Figure 4D), as well as increased expression of metastasis related protein (MMP‐9, Figure 4D). Collectively, above findings were according with our results in vitro; knockdown of SNHG7 could impede hepatic carcinoma cell proliferation in nude mice.

## 4 DISCUSSION

Numerous studies have confirmed that lncRNA exerted complex effects in progression of tumourigenesis and numerous overexpressed lncRNA has been found in hepatic carcinoma.^23^, ^24^ SNHG7 has been identified as an oncogene in breast cancer, lung cancer, and colorectal cancer.^13^, ^14^ In this work, a significant SNHG7 overexpression was observed in hepatic carcinoma tissues compared with adjacent histologically normal hepatic tissues. In addition, overexpressed SNHG7 in hepatic carcinoma patients was significantly associated with aggressiveness, OS rate, and clinical stage. Knockdown experiments identified the oncogenous function of SNHG7 in promoting cellular proliferation and metastasis, suppressing apoptosis in hepatic carcinoma. Furthermore, in vivo assay revealed that silence of SNHG7 could inhibit tumour growth. Meanwhile, immunohistochemical assay illuminated knockdown of SNHG7 promoted cell apoptosis in tumour tissues, as well as suppressed metastasis of hepatic carcinoma.

ceRNA has emerged as an alternative function for lncRNAs.^25^ lncRNA can interact with the miRNA directly to regulate the expression of target genes. Novel regulatory network has been identified in crosstalk between lncRNAs and mRNAs.^26^ lncRNA SNHG7 has been recognized as a sponge of miR‐216b to promote proliferation and liver metastasis of colorectal cancer via upregulating GALNT1.^14^ Bioinformatics analysis was conducted to reveal putative binding miRNA of SNHG7; putative binding sites was found between SNHG7 and miR‐425. Following that, it was proved that there was an endogenous interaction between miR‐425 and SNHG7 by performing the dual‐luciferase assay. Subsequently, rescued miR‐425 was observed when SNHG7 was silenced, which further confirmed SNHG7 sponging miR‐425 as a ceRNA.

WNT/β‐catenin pathway plays a crucial role in regulation process of cell proliferation, differentiaton, and tumourigenesis, and it has been recognized as a target of ncRNA in cancer proliferation and EMT of carcinogenesis.^17^ Further molecular biology experiment illustrated that the protein levels of β‐catenin, MMP‐9, and N‐cadherin, which could promote carcinogenesis, were decreased markedly; by contrary, metastatic suppress protein E‐cadherin was increased notably both in miR‐425‐mimic or SNHG7‐siRNA transfected HepG2 and HCC‐LM3 cells. The above results suggested that SNHG7 could promote proliferation and metastasis of hepatic carcinoma cell via Wnt/β‐catenin/EMT pathway via sponging miR‐425.

Collectively, SNHG7 could promoted proliferation and metastasis of hepatic carcinoma cell in vitro and in vivo, suggesting that SNHG7 exert tumorigenic role in hepatic carcinoma progression. Further mechanism research revealed that SNHG7 exhibited the tumorigenic role through Wnt/β‐catenin/EMT pathway as a miR‐425 sponge. These findings provided new cues to understand the molecular signalling network in carcinogenesis of hepatic carcinoma, and it may provide new evidence for therapeutic application in hepatic carcinoma.

## AVAILABILITY OF DATA AND MATERIALS

The data sets used or analysed in this study are available from the corresponding author on reasonable request.

## ETHICS APPROVAL AND CONSENT TO PARTICIPATE

Tissues and information of patients were gained with written informed consent. All of the animal experiments in this study were performed according to the National Institutes of Health (Bethesda, MD, USA) guidelines and were approved by the Institutional Animal Care and Treatment Committee of Second Affiliated Hospital of Nanchang University. Procedures involving patients in this study were approved by the Human Ethics Committee at the Second Affiliated Hospital of Nanchang University.

## CONSENT FOR PUBLICATION

Consent for the publication of the clinical and pathological data was obtained from all patients who were involved in this study.

## CONFLICT OF INTEREST

Authors declare that they have no competing interests.

## AUTHOR CONTRIBUTIONS

X.Y. and Z.G. designed the study. X.Y. and C.L. wrote the article. X.Y., C.L., W.X., and C.L. collected and analysed the data. Z.G. collected and analysed the data to revise the manuscript in accordance with reviewer's comments. Also, S.S. helped the authors write the revised version. All authors read and approved the final manuscript.

## FUNDING INFORMATION

This work was supported by the Science and Technology Research Project of Education Department of Jiangxi Province (no. GJJ170099).

## References

[1] Kim NG , Nguyen PP , Dang H , et al. Temporal trends in disease presentation and survival of patients with hepatocellular carcinoma: a real‐world experience from 1998 to 2015. Cancer. 2018;124(12):2588‐2598. 29624631 10.1002/cncr.31373

[2] Poon D , Anderson BO , Chen L‐T , et al. Management of hepatocellular carcinoma in Asia: consensus statement from the Asian Oncology Summit 2009. Lancet Oncol. 2009;10(11):1111‐1118. 19880065 10.1016/S1470-2045(09)70241-4

[3] Aravalli RN , Cressman ENK , Steer CJ . Cellular and molecular mechanisms of hepatocellular carcinoma: an update. Arch Toxicol. 2013;87(2):227‐247. 23007558 10.1007/s00204-012-0931-2

[4] Aravalli RN , Steer CJ , Cressman ENK . Molecular mechanisms of hepatocellular carcinoma. Hepatology. 2008;48(6):2047‐2063. 19003900 10.1002/hep.22580

[5] Sia D , Villanueva A , Friedman SL , Llovet JM . Liver cancer cell of origin, molecular class, and effects on patient prognosis. Gastroenterology. 2017;152(4):745‐761. 28043904 10.1053/j.gastro.2016.11.048 PMC12160040

[6] Anastasiadou E , Jacob LS , Slack FJ . Non‐coding RNA networks in cancer. Nat Rev Cancer. 2018;18(1):5‐18. 29170536 10.1038/nrc.2017.99 PMC6337726

[7] Taft RJ , Pang KC , Mercer TR , Dinger M , Mattick JS . Non‐coding RNAs: regulators of disease. J Pathol. 2010;220(2):126‐139. 19882673 10.1002/path.2638

[8] X‐y F , H‐f P , R‐x L , Ye D‐q . Long noncoding RNAs: novel insights into gastric cancer. Cancer Lett. 2015;356(2):357‐366. 25444905 10.1016/j.canlet.2014.11.005

[9] Liz J , Esteller M . lncRNAs and microRNAs with a role in cancer development. Biochim Biophys Acta Gene Regul Mech. 2016;1859(1):169‐176. 10.1016/j.bbagrm.2015.06.015 26149773

[10] Prensner JR , Chinnaiyan AM . The emergence of lncRNAs in cancer biology. Cancer Discov. 2011;1(5):391‐407. 22096659 10.1158/2159-8290.CD-11-0209 PMC3215093

[11] Tay Y , Rinn J , Pandolfi PP . The multilayered complexity of ceRNA crosstalk and competition. Nature. 2014;505(7483):344‐352. 24429633 10.1038/nature12986 PMC4113481

[12] Li J‐H , Liu S , Zhou H , Qu L‐H , Yang J‐H . starBase v2.0: decoding miRNA‐ceRNA, miRNA‐ncRNA and protein‐RNA interaction networks from large‐scale CLIP‐Seq data. Nucleic Acids Res. 2014;42(D1):D92‐D97. 24297251 10.1093/nar/gkt1248 PMC3964941

[13] Li Y , Zeng C , Hu J , et al. Long non‐coding RNA‐SNHG7 acts as a target of miR‐34a to increase GALNT7 leve and regulate PI3K/Akt/mTOR pathway in colorectal cancer progression. J Hematol Oncol. 2018;11(1):89. 29970122 10.1186/s13045-018-0632-2 PMC6029165

[14] Shan Y , Ma J , Pan Y , Hu J , Liu B , Jia L . LncRNA SNHG7 sponges miR‐216b to promote proliferation and liver metastasis of colorectal cancer through upregulating GALNT1. Cell Death Dis. 2018;9(7):722. 29915311 10.1038/s41419-018-0759-7 PMC6006356

[15] She K , Huang J , Zhou H , Huang T , Chen G , He J . lncRNA‐SNHG7 promotes the proliferation, migration and invasion and inhibits apoptosis of lung cancer cells by enhancing the FAIM2 expression. Oncol Rep. 2016;36(5):2673‐2680. 27666964 10.3892/or.2016.5105

[16] She K , Yan H , Huang J , Zhou H , He J . miR‐193b availability is antagonized by LncRNA‐SNHG7 for FAIM2‐induced tumour progression in non‐small cell lung cancer. Cell Prolif. 2018;51(1). 10.1111/cpr.12406 PMC6528931 29131440

[17] Clevers H , Nusse R . Wnt/beta‐catenin signaling and disease. Cell. 2012;149(6):1192‐1205. 22682243 10.1016/j.cell.2012.05.012

[18] Ying Y , Tao Q . Epigenetic disruption of the WNT/beta‐catenin signaling pathway in human cancers. Epigenetics. 2009;4(5):307‐312. 10.4161/epi.4.5.9371 28001119

[19] Brabletz T , Kalluri R , Angela Nieto M , Weinberg RA . EMT in cancer. Nat Rev Cancer. 2018;18(2):128. 29326430 10.1038/nrc.2017.118

[20] Ghahhari NM , Babashah S . Interplay between microRNAs and WNT/beta‐catenin signalling pathway regulates epithelial‐mesenchymal transition in cancer. Eur J Cancer. 2015;51(12):1638‐1649. 26025765 10.1016/j.ejca.2015.04.021

[21] Li D , Tian B , Jin X . miR‐630 inhibits epithelial‐to‐mesenchymal transition (EMT) by regulating Wnt/betacatenin pathway in gastric cancer cells. Oncol Res. 2018;27(1):9‐17. 29422112 10.3727/096504018X15178732625479 PMC7848419

[22] Ma F , Li W , Liu C , et al. MiR‐23a promotes TGF‐beta 1‐induced EMT and tumor metastasis in breast cancer cells by directly targeting CDH1 and activating Wnt/beta‐catenin signaling. Oncotarget. 2017;8(41):69538‐69550. 29050223 10.18632/oncotarget.18422 PMC5642498

[23] Klingenberg M , Matsuda A , Diederichs S , Patel T . Non‐coding RNA in hepatocellular carcinoma: mechanisms, biomarkers and therapeutic targets. J Hepatol. 2017;67(3):603‐618. 28438689 10.1016/j.jhep.2017.04.009

[24] Li S , Huang Y , Huang Y , et al. The long non‐coding RNA TP73‐AS1 modulates HCC cell proliferation through miR‐200a‐dependent HMGB1/RAGE regulation. J Exp Clin Cancer Res. 2017;36(1):51. 28403886 10.1186/s13046-017-0519-z PMC5389141

[25] Cesana M , Cacchiarelli D , Legnini I , et al. A long noncoding rna controls muscle differentiation by functioning as a competing endogenous RNA. Cell. 2011;147(2):358‐369. 22000014 10.1016/j.cell.2011.09.028 PMC3234495

[26] Shi X , Sun M , Liu H , Yao Y , Song Y . Long non‐coding RNAs: a new frontier in the study of human diseases. Cancer Lett. 2013;339(2):159‐166. 23791884 10.1016/j.canlet.2013.06.013

